# Fitness of Spontaneous Rifampicin-Resistant *Staphylococcus aureus* Isolates in a Biofilm Environment

**DOI:** 10.3389/fmicb.2019.00988

**Published:** 2019-05-07

**Authors:** Lisa Maudsdotter, Yuri Ushijima, Kazuya Morikawa

**Affiliations:** Department of Biomedical Science, University of Tsukuba, Tsukuba, Japan

**Keywords:** *Staphylococcus aureus*, biofilm, rifampicin, fitness, resistance

## Abstract

Biofilms of *S. aureus* accumulate cells resistant to the antibiotic rifampicin. We show here that the accumulation of rifampicin resistant mutants (RifR) in biofilms is not equable but rather is a local event, suggesting that the growth of a few locally emerged mutants is responsible for this. Competition assays demonstrated that, compared to wild-type bacteria, the isolated RifR mutants have a growth advantage in biofilms, but not in planktonic culture. To gain insight into the mechanism of the growth advantage, we tested the involvement of the two-component systems (TCS) that sense and respond to environmental changes. We found that a deletion of SrrAB or NreBC has a drastic effect on the growth advantage of RifR mutants, suggesting the importance of oxygen/respiration responses. All six of the RifR isolates tested showed increased resistance to at least one of the common stresses found in the biofilm environment (i.e., oxidative, nitric acid, and organic acid stress). The RifR mutants also had a growth advantage in a biofilm flow model, which highlights the physiological relevance of our findings.

## Introduction

*Staphylococcus aureus* is an opportunistic human pathogen responsible for diverse infectious diseases (Lowy, 1998; Wertheim et al., 2005). *S. aureus* biofilm-associated infections are especially difficult to treat by antibiotics since their penetration into the structure and access to the bacterial cells is inhibited, thus a series of preventative and treatment strategies have been tested (Suresh et al., 2018). Implanted materials, such as catheters or cannula, often get coated in biomolecules which can play a role in facilitating attachment, and subsequent development of biofilms, of *S. aureus* (Khatoon et al., 2018). The biofilm matures through the growth of bacteria and the production of an extracellular matrix that serves as a shelter against challenges from host bactericidal factors (Otto, 2008), as well as antibiotics. A mature biofilm can also act as a reservoir of *S. aureus* cells in the body, as cells can detach from the biofilm and spread to other sites to cause recurrent infections, including bacteremia (Lister and Horswill, 2014).

Although a recent report failed to show a benefit in using rifampicin as an adjunct to standard antibiotic therapy against *S. aureus* bacteremia (Thwaites et al., 2018), rifampicin is one of the antibiotics that can efficiently penetrate the biofilm of *S. aureus* (Raad et al., 2007), and the use of rifampicin on biofilm-related infections has long been considered rational (Zimmerli and Sendi, 2019). On the other hand, *S. aureus* grown in biofilms accumulates rifampicin resistance. The emergence of rifampicin resistance has been suggested to be a consequence of oxidative stress-induced mutagenesis (Ryder et al., 2012).

In general, an increase in frequency of antibiotic resistant mutants depends largely on how the mutation influences the bacterial fitness, i.e., the ability to replicate and survive in a competitive environment. An important aspect is that a mutation may have different impacts on the bacterial growth and/or viability in different environments. In this study, we speculated that the accumulation of rifampicin resistant mutants over time might be attributed to certain fitness advantages in addition to an increased mutation rate. Hence, we investigated the fitness of spontaneous rifampicin resistant mutants in biofilm environment. We found that spontaneous rifampicin resistant mutants had a growth advantage in the biofilm environment but not in liquid cultures. Further, the rifampicin resistant mutants had increased resistance to stress conditions encountered in a biofilm environment, such as oxidative and lactate stress.

## Materials and Methods

### Bacterial Strains and Growth Conditions

*Staphylococcus aureus* strains (listed in Table 1) were cultured in brain heart infusion (BHI) broth (Becton Dickinson and Company, MD, United States) for approx. 16–20 h at 37°C with shaking, before experiments. For bacterial enumeration, BHI plates with 1.5% agar (Wako Pure Chemical Industries, Ltd., Japan) were used.

**Table 1:** *S. aureus* strains and primers used in this study.

Strain	Description	rifampicin MIC^∗^	*rpoB* mutation	Source
N315	Pre-MRSA, Km^R^	≤0.0078	wild -type^∗∗^	Kuwahara-Arai et al., 1996
RifRl	Rifampicin resistant mutant of N315, from biofilm	>8	substitution: A_477_D	This study
RifR2	Rifampicin resistant mutant of N315, from biofilm	>8	substitution: A_477_D	This study
RifR3	Rifampicin resistant mutant of N315, from biofilm	>8	none^∗∗∗^	This study
RifR4	Rifampicin resistant mutant of N315, from biofilm	8	none^∗∗∗^	This study
RifR5	Rifampicin resistant mutant of N315, from biofilm	1	S 486 A 487 to S 486 [L, S] A487	This study
RifR6	Rifampicin resistant mutant of N315, from biofilm	1	S 486 A 487 to S 486 [L, S] A487	This study
RifRliql	Rifampicin resistant mutant of N315, from liquid culture			This study
RifRliq2	Rifampicin resistant mutant of N315, from liquid culture			This study
N315ex	SCCmec cured derivative of N315, Km^S^			Ito et al., 1999
deltaSigB	*sigB* deletion mutant of N315			This study

**Primers**	**5′ - 3′**			

SA1869fl	AAGAATTCCCTACAGTTATTGTTGCGGC			This study
RsbU2	TACCCGGGCAATTTTGCTGTAGATGA			This study
SA1869rl	TGCCCGGGCCATATAATTATCCCTTGA			This study
SA1869r2	CTAGATCTGAACTTCATCTAGTCCACCAGT			This study
rpoB-F	AATATAGAATCGAAAATGGTGTCAT			This study
rpoB-R	CCTTCAATTTAGATTAGCTGTGCTAT			This study

*^∗^MIC was measued in BHI medium. ^∗∗^GenBank accession: BA000018.3. ^∗∗∗^in whole *rpoB* coding region.*

The *sigB* mutant (deltaSigB) was constructed in strain N315 by double crossover homologous recombination. Upstream and downstream regions of *sigB* were amplified by PCR using primers SA1869f1 and RsbU2 (for upstream), and SA1869r1 and SA1869r2 (for downstream) (Table 1). The two fragments were blunt-end digested by *Sma* I and ligated together. The ligate was amplified using primers SA1869f1 and SA1869r2 and cloned into the *Eco*R I – *Bgl* II site of pMAD-tet (Morikawa et al., 2012). The pMAD plasmid carries a temperature sensitive replication origin and the β-galactosidase gene for color selection, which facilitate the mutant isolation (Arnaud et al., 2004). Briefly, N315 carrying the targeting vector inserted in its genome was selected at a non-permissive temperature in the presence of tetracycline (5 μg/ml). The *sigB*-deleted mutants generated through the plasmid excision event were selected as white colonies on agar plates containing 50 μg/mL X-gal. The absence of the *sigB* gene was confirmed by PCR.

Deletion mutants of each TCS (except the essential WalRK which regulates cell wall metabolism (Dubrac and Msadek, 2004; Dubrac et al., 2007), and TCS2 in SCCmec) were constructed from N315ex (to be published elsewhere). Briefly, the upstream and downstream regions of the target locus were amplified by PCR and inserted into the *Bam*H I – *Sal* I site of pMAD-tet. Mutants were selected and confirmed as described above.

### Cellulose Disk Biofilm Model

Mixed cellulose-ester membrane filters (25 mm diameter, 0.22 μm pore size; Millipore) were coated with human plasma and inoculated with *S. aureus* as previously described (Ryder et al., 2012). Briefly, membranes were incubated in 4% normal pooled human plasma (Sigma-Aldrich, St. Louis, MO, United States) diluted in 0.05 M carbonate buffer at 4°C overnight. The plasma-coated disks were soaked in overnight cultures of *S. aureus*, placed on BHI agar and incubated at 37°C. After incubation the membranes were placed in 1 ml 0.9% NaCl, vigorously vortexed for 1 min, and incubated for 30 min. The bacterial suspensions were serially diluted and plated for enumeration. In some experiments the membranes were cut into 14 sections by sterile scissor and tweezers before being placed in NaCl, as previously described (Wrande et al., 2008). To isolate spontaneous rifampicin resistant mutants, biofilms of N315 were incubated for 1 day. Thereafter bacteria were recovered as above and spread on BHI agar plates containing 3 μg/ml rifampicin.

### Survival in Biofilm

Biofilms of N315ex (kanamycin sensitive) were grown on cellulose-ester membranes as above and incubated for 2 days at 37°C. N315 wild-type and mutants were introduced to the center of the biofilm at number of approx. 10,000 cfu in 2 μl. The biofilms were then incubated for 7 days at 37°C. Bacteria were recovered as described above and N315 wild-type and mutants were quantified on BHI plates containing 50 μg/ml kanamycin.

### Survival in Stationary Liquid Cultures

N315 wild-type and mutants (10,000 cfu in 2 μl) were introduced to 1 day old stationary phase N315ex cultures grown in BHI medium. The cultures were then incubated for 7 days at 37°C. Survivors were enumerated by plating serial dilutions on BHI plates containing 50 μg/ml kanamycin.

### Quantification of Nitrite

Bacteria were incubated in nitrate broth (5 g/l peptone, 3 g/l beef extract, 3.36 g/l sodium nitrate), or in nitrite broth (peptone, beef extract, 1 mM sodium nitrite: added just prior to use) at 37°C. Nitrite was quantified by conventional Griess reaction following the method described in the manufacture’s instruction (Griess Reagent Kit for Nitrite Determination (G-7921), Molecular Probes, Inc.). At different time points 100 μl culture was transferred to a 96 well plate to which 50 μl of 46.2 mM sulfanic acid dissolved in 5N acetic acid and 50 μl of 41.9 mM N-1-naphylehylene diamine dihydrochloride dissolved in 5N acetic acid were added. A red color development indicates presence of nitrite. Absorbance was measured at 548 nm. The concentrations were calculated by the equation from the plot of standard samples.

### Bacterial Growth in Organic Acids

Bacterial growth in BHI broth supplemented with organic acids was analyzed by measuring the optical density at 600 nm, every 15 min for 16 h. Overnight cultures were diluted 1:100 and added to the wells of a 96 well plate with a final volume of 300 μl. Lactic acid (Sigma), acetic acid, and formic acid were each used at the concentrations of 1 and 10 mM.

### Nitrogen Oxide Killing Assay

S-Nitroso-N-acetyl-DL-penicillamine (SNAP; Sigma-Aldrich, St. Louis, MO, United States) was used as nitric oxide (NO) source (Kaplan et al., 1996). *S. aureus* cells were harvested from overnight cultures and washed with 0.9% NaCl. Thirty μl equivalent of *S. aureus* cells suspended in 270 μl of 0.9% NaCl were challenged with ⋅NO by the addition of 30 μl of 1 mM SNAP and incubation for 24 h.

### H_2_O_2_ Killing Assay

The oxidative stress assay was performed as described previously (Ushijima et al., 2014). Briefly, 40 μl of OD_600_ 10 equivalent *S. aureus* cells were harvested from overnight culture, washed once with ice-cold PBS and suspended in 400 μl ice-cold PBS. Fifty μl of *S. aureus* cells were suspended in 450 μl of PBS with or without 400 mM H_2_O_2_ (final concentration) and incubated at RT for 3 min.

### Flow Biofilm Model

The Vena8 Biochip (Cellix) was assembled and coated with 4% human plasma diluted in 0.05 M carbonate buffer overnight. N315ex (20 ul overnight culture) was added to each well and the biochip was incubated at room temperature for 1 h. The biochip was connected to a pump and incubated at 37°C with a flow of 50 μl BHI /min for 1 day. N315 wild-type and mutants were introduced to the center of the biofilm at a number of approx. 10^7^ cfu in 20 μl and the biochip was incubated at room temperature for 1 h. The biofilms were then incubated for 7 days at 37°C with a flow of 50 μl/min. After incubation the biochip bottom plate was removed and the biofilms were scraped off with a pipette tip and collected. Bacteria were enumerated by plating onto BHI plates containing 50 μg/ml kanamycin.

### Sequencing of *rpoB*

The *rpoB* gene, including the entire coding region, was amplified by PCR with primers rpoB-F and rpoB-R. The PCR products were purified and the entire coding regions were sequenced (FASMAC, Japan).

### MIC Measurement

Minimum inhibitory concentrations (MICs) of rifampicin were determined by micro-dilution method according to the CLSI standard procedure using BHI broth.

## Results

### Spontaneous RifR Mutants Have Increased Fitness in Biofilm

**FIGURE 1:**
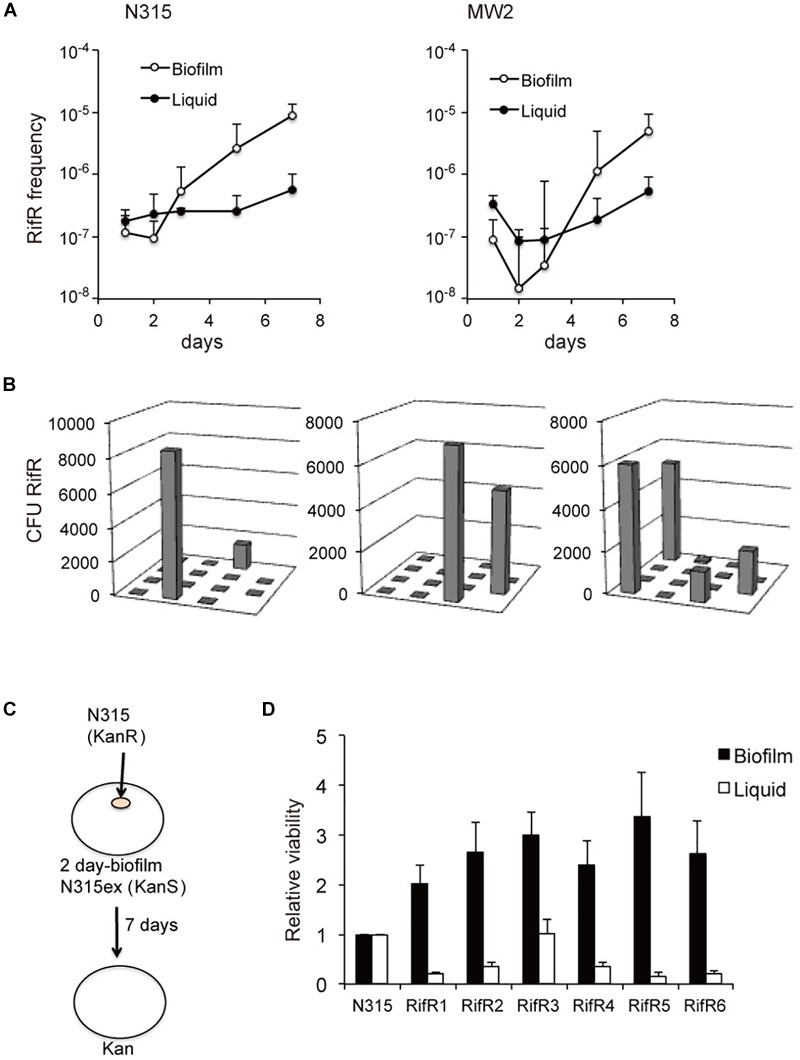
Increased fitness of spontaneous RifR mutants in biofilm. **(A)** Aging biofilms of *S. aureus* accumulate RifR cells. **(B)** Distribution of the RifR cells in the biofilm. The biofilm disc was cut in sections and RifR cells were counted. **(C,D)** Fitness of the RifR mutants in biofilm environment. A small number of RifR mutants (kanamycin resistant) were inoculated in the center of a two-day-old biofilm of kanamycin sensitive bacteria (N315ex) and incubated for 7 days. RifR mutants (RifR1 ∼6) had increased viability in biofilm in comparison to the wild-type (N315).

We investigated the nature of rifampicin resistant cells (RifR) that accumulated in *S. aureus* biofilms using a plasma coated cellulose disk biofilm model as previously described (Ryder et al., 2012). In accordance with the previous study (Ryder et al., 2012), aging biofilms of *S. aureus* accumulated RifR cells (Figure 1A,B). The phenomenon of accumulating RifR cells did not occur in aging liquid cultures. RifR accumulation was previously suggested to be a consequence of oxidative stress-induced mutagenesis of biofilm-grown *S. aureus* (Ryder et al., 2012). We speculated that an increased fitness of RifR cells in the biofilm environment might also contribute to this accumulation in a manner similar to that previously observed for RifR cells in aging colonies of the Gram-negative bacteria *Salmonella* and *Escherichia coli* (Wrande et al., 2008; Katz and Hershberg, 2013). We analyzed the distribution of the RifR cells in the biofilm by cutting the biofilm in sections and plating each section individually, as previously done for *Salmonella* (Wrande et al., 2008). The RifR cells have a clustered distribution (Figure 1B), which suggests that the accumulation originated from the growth of a few mutants that emerged in specific localities. Next, we isolated six different RifR cells from one-day-old biofilms grown without antibiotic selection (RifR1 ∼ RifR6). The *rpoB* sequences and rifampicin MIC values are shown in Table 1. To test the fitness of the RifR mutants in the biofilm environment we introduced a small number of mutant or N315 wild-type bacteria (kanamycin resistant) in the center of a two-day-old biofilm of strain N315ex (kanamycin sensitive) and incubated the biofilm for 7 days (Figure 1C). N315ex is a derivative of N315 that has lost the SCCmec carrying the kanamycin (aminoglycoside) resistance gene. The two-day time point was chosen for inoculation as the biofilm model usually shows accumulation of RifR between 3 and 7 days of growth. All six RifR mutants tested had increased growth in the biofilm in comparison to the wild-type (Figure 1D). The same RifR mutants were also tested for growth in stationary phase liquid culture using a similar approach; introducing mutant or wild-type as a minority in a stationary phase liquid culture and incubating for 7 days. Strikingly, in planktonic culture, none of the RifR mutants had greater viability than the wild-type. We also tested two rifampicin resistant mutants isolated from stationary phase liquid cultures for fitness in biofilm. These two RifR mutants also had a growth advantage over the wild-type in a biofilm environment (Supplementary Figure S1). In conclusion, spontaneous RifR mutants have a growth advantage in aging biofilms but not in liquid cultures.

### Mutants With Defective Nitrate and Nitrite Reduction Show Increased Accumulation of RifR Mutants

**FIGURE 2:**
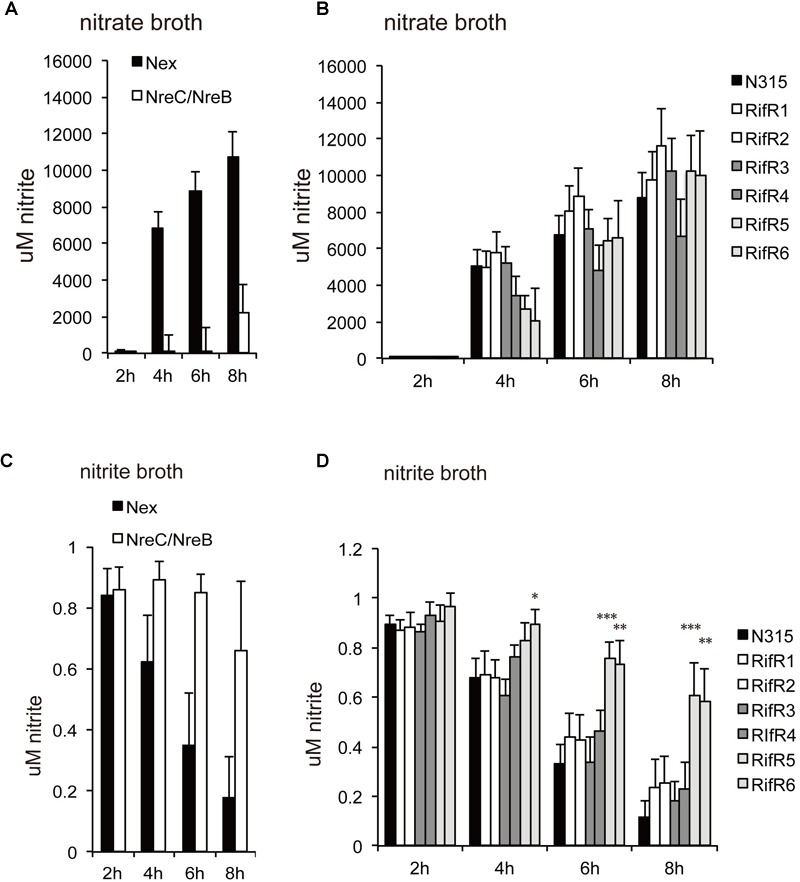
Nitrate (NO^3-^) and nitrite (NO^2-^) reduction. **(A,B)** Cells were grown in nitrate broth. **(C,D)** Cells were grown in nitrite broth. **(A,C)** ΔNreB/C. **(B,D)** Rifampicin resistant mutants. The average concentrations of nitrite were shown with standard deviations. Two of the RifR mutants (RifR5 and RifR6) had reduced nitrite reduction ability. ^∗∗∗^*p* < 0.01, ^∗∗^*p* < 0.05, ^∗^*p* < 0.1, in *t*-test.

*S. aureus* has several systems to sense and respond to its environment. A deletion mutant of the general stress responsive sigma factor, SigB, had reduced viability in biofilm (Supplementary Figure S2A). However, biofilms of the SigB deletion mutant still accumulated rifampicin resistant cells over time (Supplementary Figure S2B). Thus, the SigB controlled stress response is not a requisite for the growth advantage of the RifR mutants. *S. aureus* carries 17 two-component systems (TCS), which sense and respond to environmental changes (Morikawa et al., 2010). We generated biofilms of a set of TCS deletion mutants and quantified the number of RifR mutants after 7 days incubation. Interestingly, we found altered RifR accumulation in two oxygen-responsive systems: SrrAB (staphylococcal respiratory response AB, aka SrhSR) and NreBC (nitrogen regulation BC). SrrAB is an ortholog of *Bacillus subtilis* ResDE, and is important for *S. aureus* ability to grow under anaerobic conditions (Throup et al., 2001; Yarwood et al., 2001). NreBC is an oxygen-responsive nitrogen regulation system (Schlag et al., 2008). The ΔSrrA/B mutant contained very few RifR cells (Supplementary Figure S2C). On the contrary, the ΔNreB/C mutant contained increased number of RifR cells (Supplementary Figure S2C). The increased number of RifR cells in biofilms of ΔNreB/C could result both from a potential increased mutation rate of the ΔNreB/C mutant or from a fitness increase of the RifR mutants. We sectioned ΔNreB/C biofilms to check the distribution of the RifR cells as previously done for the wild-type. Similar to the wild-type biofilm, the RifR cells in the ΔNreB/C biofilm were clustered in a few sections of the biofilm (Supplementary Figure S2D). Deletion of NreABC was previously reported to have reduced expression of nitrate assimilation genes, and impaired reduction of nitrate (NO_3_^-^) and nitrite (NO_2_^-^) (Schlag et al., 2008). Consistent with this, our ΔNreB/C mutant was impaired in nitrate (Figure 2A) and nitrite reduction (Figure 2C). This data urged us to investigate nitrate and nitrite reduction of the rifampicin resistant mutants. None of the mutants were affected in nitrate reduction (Figure 2B). However, two of the RifR mutants (RifR5 and RifR6) had reduced nitrite reduction ability, while others (RifR1 ∼RifR4) had no significant difference from the wild type (Figure 2D).

### The Reduced Relative Viability of RifR Mutants in Aging Liquid Cultures Is Abolished by the Addition of Nitrate or Nitrite

**FIGURE 3:**
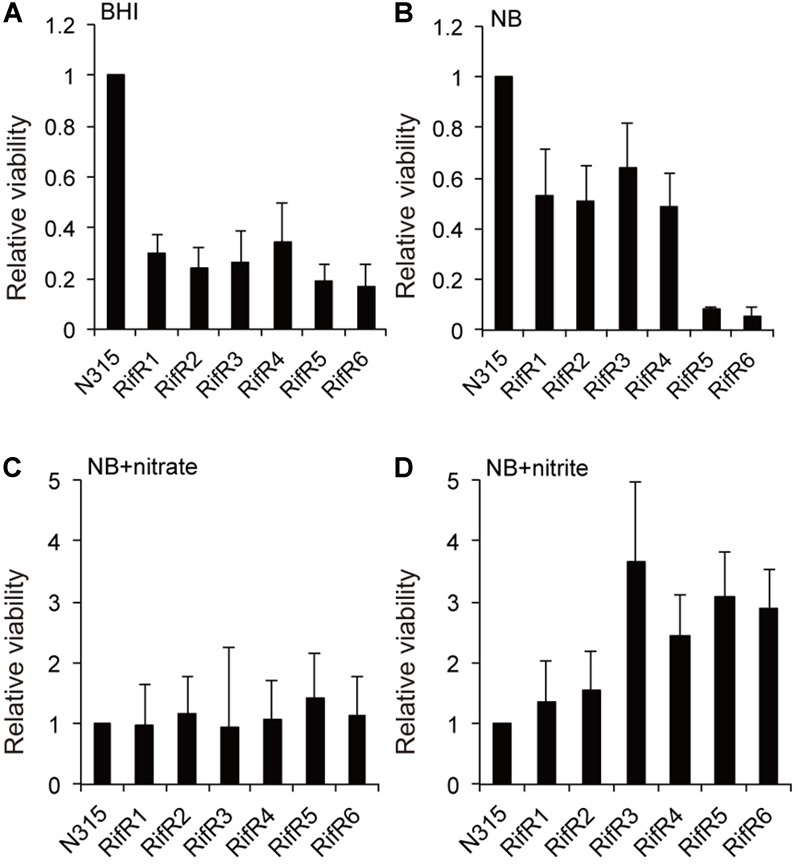
Effect of the addition of nitrate or nitrite on the reduced viability of RifR mutants in aging liquid cultures. **(A,B)** The RifR mutants had reduced viability in aging liquid cultures in brain-heart infusion (BHI) medium **(A)** or in nutrient broth (NB) **(B)**. **(C)** Addition of 40 mM nitrate diminishes the viability reduction in NB. **(D)** 1 mM nitrite confers increased viability to RifR cells relative to the wild-type. Mean relative viability and standard deviations compared to N315 is shown in all the graphs (*n* = 3).

The relative viability of the RifR mutants in stationary liquid culture was tested by a competition assay as described in the Methods section. The six RifR mutants had reduced viability in aging liquid cultures when grown in the rich brain-heart infusion medium (Figure 3A). However, for four of the mutants the reduced viability was less pronounced when cultured in the less-rich nutrient broth (NB) medium (5 g/l peptone, 3 g/l beef extract) (Figure 3B). For all the rifampicin resistant mutants the difference in viability compared to the wild-type was abolished when 40 mM nitrate was added to the liquid medium (Figure 3C). Strikingly, for four of the RifR mutants, an addition of 1 mM nitrite to the NB broth caused an increased viability in comparison to the wild-type (Figure 3D). Since addition of nitrate or nitrite did not affect the relative viability of the wild type (N315) in the background strain (N315ex), these results suggest that the rifampicin resistant mutants are more tolerant to the nitrogen intermediate species. Thus, the relative fitness of rifampicin resistant mutants is environment dependent.

The presence of nitrite in the biofilm was quantified to be approx. 5∼15 nmol/disk irrespective of biofilm age (1–7 days). Given that the volume of biofilm per disk is about 30 μl, the estimated concentration is about 0.2∼0.5 mM. This value is a mean of the entire biofilm and the nitrite concentration might be locally different.

### Stress Resistance of RifR Mutants

**FIGURE 4:**
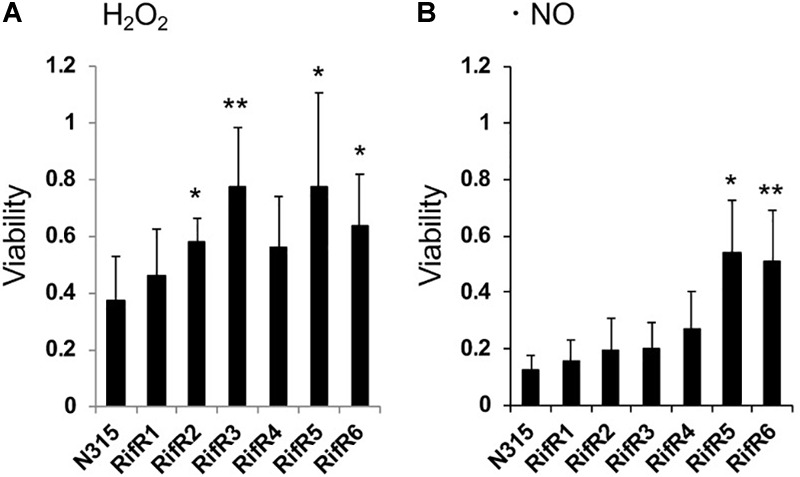
Resistance of rifampicin resistant mutants to hydrogen peroxide and nitric oxide. **(A)** Hydrogen peroxide (H_2_O_2_). **(B)** Nitric oxide (NO). Means and standard deviations of four **(A)** or three **(B)** independent experiments are shown. ^∗∗^*p* < 0.05, ^∗^*p* < 0.1, in *t*-test.

**FIGURE 5:**
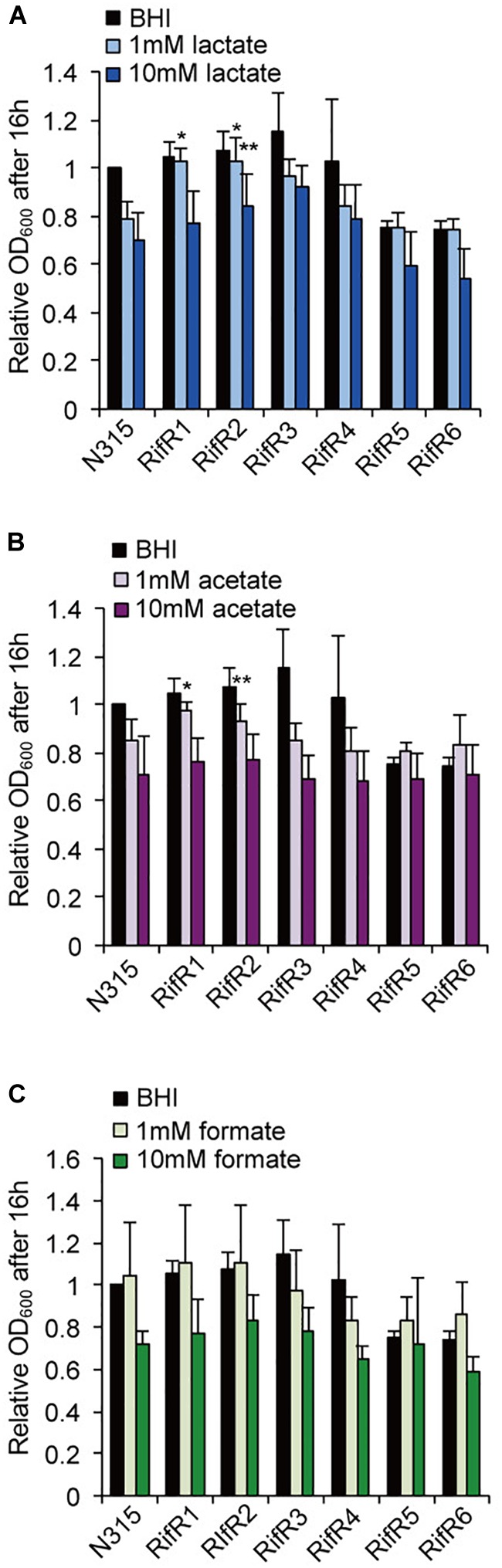
Effects of organic acids on the growth of rifampicin resistant mutant. **(A)** Lactic acid, **(B)** Acetic acid, **(C)** Formic acid. The acids concentrations were 1 mM or 10 mM. Mean and standard error values of bacterial growth yields (OD_600_ after 16 h) are shown (*n* = 3). ^∗∗^*p* < 0.05, ^∗^*p* < 0.1, in *t*-test. See Supplementary Figure S3 for growth curves.

We tested the resistance of the rifampicin resistant mutant to nitric oxide (NO) and hydrogen peroxide (H_2_O_2_). Some RifR mutants were significantly more resistant to H_2_O_2_ (Figure 4A) and nitric oxide (Figure 4B). *S. aureus* grown in biofilm catabolizes glucose and accumulates the organic acids lactate, acetate, and formate (Zhu et al., 2007). We tested the effects of these organic acids on the growth of *S. aureus* wild-type and rifampicin resistant mutants (Figure 5 and Supplementary Figure S3). The acids were used at the concentration of 1 mM and 10 mM, which is within the range found to accumulate in biofilms (Zhu et al., 2007). For the wild-type, addition of 1 mM lactate or acetate reduced the growth (Supplementary Figure S3). Interestingly, the growth curves of RifR1 and RifR2 were not affected by 1mM lactate (Supplementary Figure S3), and they showed significantly higher OD_600_ after 16h growth compared with wild type (Figure 5A). RifR1 and RifR2 were also tolerant for 1 mM acetate (Figure 5B and Supplementary Figure S3). In terms of formate, no statistically significant increase was observed in any RifR mutant. For RifR5 and RifR6, growth in BHI is impaired and effect of organic acids was less compared with wild type and other RifR mutants.

### RifR Mutants Have Improved Fitness in a Flow-Model Biofilm

**FIGURE 6:**
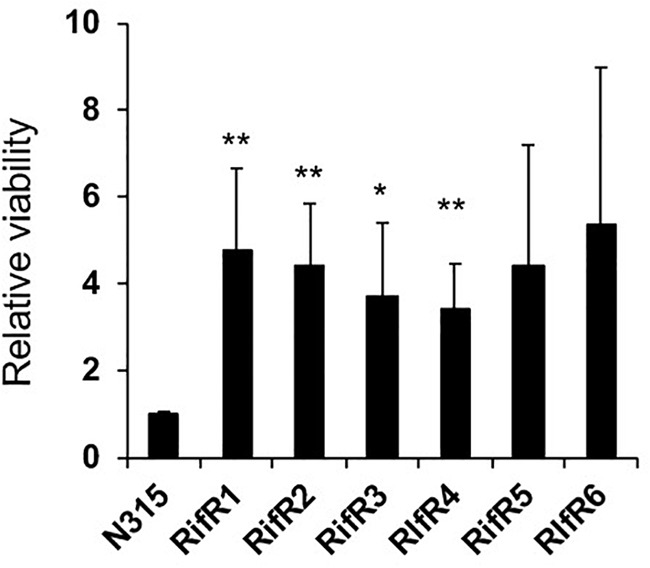
High viability of RifR mutants in biofilm under flow. Mean and standard error values of relative viability are shown (*n* = 3). ^∗∗^*p* < 0.05, ^∗^*p* < 0.1 in *t*-test.

Infections associated with medical devices such as catheters and artificial heart valves are commonly associated with the formation of a bacterial biofilm on the surface. In this kind of environment there is an on-going flow. Thus, we investigated the fitness of RifR mutants in a biofilm model with flow. In accordance with the result of static biofilm, RifR mutants exhibited better viability in the biofilm under the flow condition (Figure 6).

## Discussion

Characterization of bacterial fitness, i.e., the ability to replicate and survive in a competitive environment, during different stages of the infection cycle is one step to find effective strategies to combat infectious diseases. In the present work we investigated the influence of RifR on *S. aureus* fitness in the biofilm environment. In accordance with literature (Ryder et al., 2012), we observed an accumulation of RifR cells in biofilms over time, but the present study found a local pattern of accumulation of RifR mutants, supporting the idea that RifR mutants have increased growth within biofilms. Indeed, all six RifR mutants tested had increased viability in biofilm conditions while they had reduced viability in aging liquid culture. RifR mutants also had better survival in the flow-model biofilm competition assay. Previously, Yu et al. (2005) reported that the RifR frequency further increases in an *in vivo* biofilm model in comparison to an *in vitro* system. Although the present study did not address *in vivo* conditions, these facts suggest that RifR mutants would have better biofilm survival in both *in vitro* and *in vivo* settings.

One mechanism for *S. aureus* to sense and respond to various stress conditions is via its TCS. We found that two different oxygen-responsive TCSs, NreBC, and SrrAB, affected RifR accumulation in opposite ways. In addition to their oxygen/respiration responses (Mullner et al., 2008; Mashruwala et al., 2017), the activity of these TCSs may also be affected by the presence of nitrite/nitrate. The NreBC system in *Staphylococcus carnosus* can respond to nitrite and nitrate, especially under anaerobic conditions. The ResDE (SrrAB ortholog in *B. subtilis*) system is also known to respond to nitrite and nitric oxide that can be spontaneously generated from nitrite (LaCelle et al., 1996; Nakano et al., 1998; Nakano, 2002). These facts suggest that the growth advantage of RifR mutants could be linked to the nitrite/nitrate response and metabolism. Indeed, for all of the RifR mutants, the relative fitness reduction in aging liquids was abolished with the addition of nitrate or nitrite (Figure 3). Two of the RifR mutants (RifR5, RifR6) had reduced nitrite reduction ability (Figure 2D), and increased tolerance against nitric oxide (Figure 4B). Other RifR mutants (RifR1∼RifR4) were similar to the wild-type in their ability to reduce nitrite (Figure 2D), and their mechanism of nitrite resistance is as yet unknown. It is interesting to note that there are nitrite induced factors responsible for resistance to oxidative and nitrosative stresses (Schlag et al., 2007). Additionally, Richardson et al identified the flavohemoprotein Hmp as a key player in NO stress resistance (Richardson et al., 2006).

The isolated RifR mutants tended to have increased resistance to hydrogen peroxide (Figure 4A). Furthermore, two RifR mutants (RifR1, RifR2) showed increased growth ability in the presence of lactate. Thus, most of the isolated RifR mutants showed an increased resistance to at least one of the biofilm-associated stresses tested, suggesting the existence of distinct mechanisms for the fitness that are not necessarily shared among RifR mutants. Hydrogen peroxide and lactate are also produced by other bacterial species that may coexist with *S. aureus* in mixed bacterial biofilms (Nair et al., 2014).

In terms of the *rpoB* sequences in the RifR mutants tested in this study, RifR1 and RifR2 had an A_477_D mutation, which is one of the previously reported rifampicin resistance mutations (Wichelhaus et al., 2002; Yu et al., 2005). RifR3, and RifR4 had no mutation in the entire *rpoB* coding region. To the best of our knowledge, this is the first case of rifampicin resistance without any mutation in *rpoB* coding sequence in staphylococci. In *Mycobacterium tuberculosis*, Jamieson et al. (2014) reported one rifampicin resistant isolate (MDR-B) in which whole-genome sequencing failed to detect any mutation in the *rpoB* coding region. RifR5 and RifR6 had two-amino acid insertion at the position 486 [S_486_ A_487_ to S_486_ (L, S) A_487_]. The MICs of rifampicin in RifR5 and RifR6 were 1 μg/ml, which is lower than the other RifR mutants (Table 1). The mutations in Rif1, 2, 5, 6 are within the rif^R^ mutation cluster which has been documented to contain a series of mutations in numerous rifampicin resistant cells. In this context, it might be worth to note that the RpoB H_481_Y mutation (in cluster I) was found to affect the expression of 361 genes (Gao et al., 2013), including genes related to stresses tested in this study: L-lactate permease (x0.46), nitric oxide reductase activation proten NorD (x3.22), and nitrate/nitrite reductase gens (narH: x0.49, narG: x0.60, nasE: x0.64, nasD: x0.440). Thus, mutations in *rpoB* link to pleiotropic effects, which seems to be a common feature in bacteria (Alifano et al., 2015).

Rifampicin resistant mutants are shown to accumulate during aging in a number of bacterial species such as *Pseudomonas, Escherichia*, and *Salmonella*. In *Salmonella* the growth advantage of RpoB and RpoS (the general stress response sigma factor) mutants in aging colonies has been attributed to the increased growth in acetate (Bergman et al., 2014). Fitness-compensatory mutations in RNA polymerase genes were also reported (Brandis et al., 2012). In our study, we cannot distinguish between the direct effect of the rifampicin resistance and of certain compensatory mutations that would counteract the disadvantages conferred by the rifampicin resistance. This study did not address whether the *rpoB* mutation itself is responsible for the observed fitness in biofilm conditions. Further study is necessary to clarify the genetic mechanism.

## Conclusion

In conclusion, this study showed that N315 rifampicin resistant mutants have better fitness in biofilm conditions, likely owing to distinct resistance mechanisms against biofilm-associated stressors. Although the present study only tested mutants derived from N315, the generally accepted idea that RifR accumulation in biofilm is due to increased mutation frequency would need to be reconsidered.

## Author Contributions

LM conceived and designed the experiments. LM, YU, and KM performed the experiments and analyzed the data. LM and KM wrote the manuscript.

## Conflict of Interest Statement

The authors declare that the research was conducted in the absence of any commercial or financial relationships that could be construed as a potential conflict of interest.

## Supplementary Material

FIGURE S1 Viability of rifampicin resistant mutants isolated from stationary phase liquid cultures (Rif liq1, Rif liq2) in biofilm. Relative viabilities compared to wild-type (N315) are shown with standard errors (*n* = 3).

FIGURE S2 Involvement of stress response systems in RifR accumulation. **(A,B)** SigB is important for viability in biofilm, but dispensable for the accumulation of RifR cells. **(C)** Relative number of RifR mutants after 7 days incubation is shown. The ΔSrrA/B mutant contained very few RifR cells, while ΔNreB/C mutant contained increased number of RifR cells. **(D)** Distribution of the RifR cells in the biofilm of ΔNreB/C.

FIGURE S3 Effects of organic acids on the growth of rifampicin resistant mutant. Growth curves of each strain in the same experiment of Figure 5.
