# Supplementary figures and images for: Fitness of Spontaneous Rifampicin-Resistant Staphylococcus aureus Isolates in a Biofilm Environment
Source: Front Microbiol. 2019 May 7;10:988. doi: 10.3389/fmicb.2019.00988 (PMC6514104; doi:10.3389/fmicb.2019.00988)

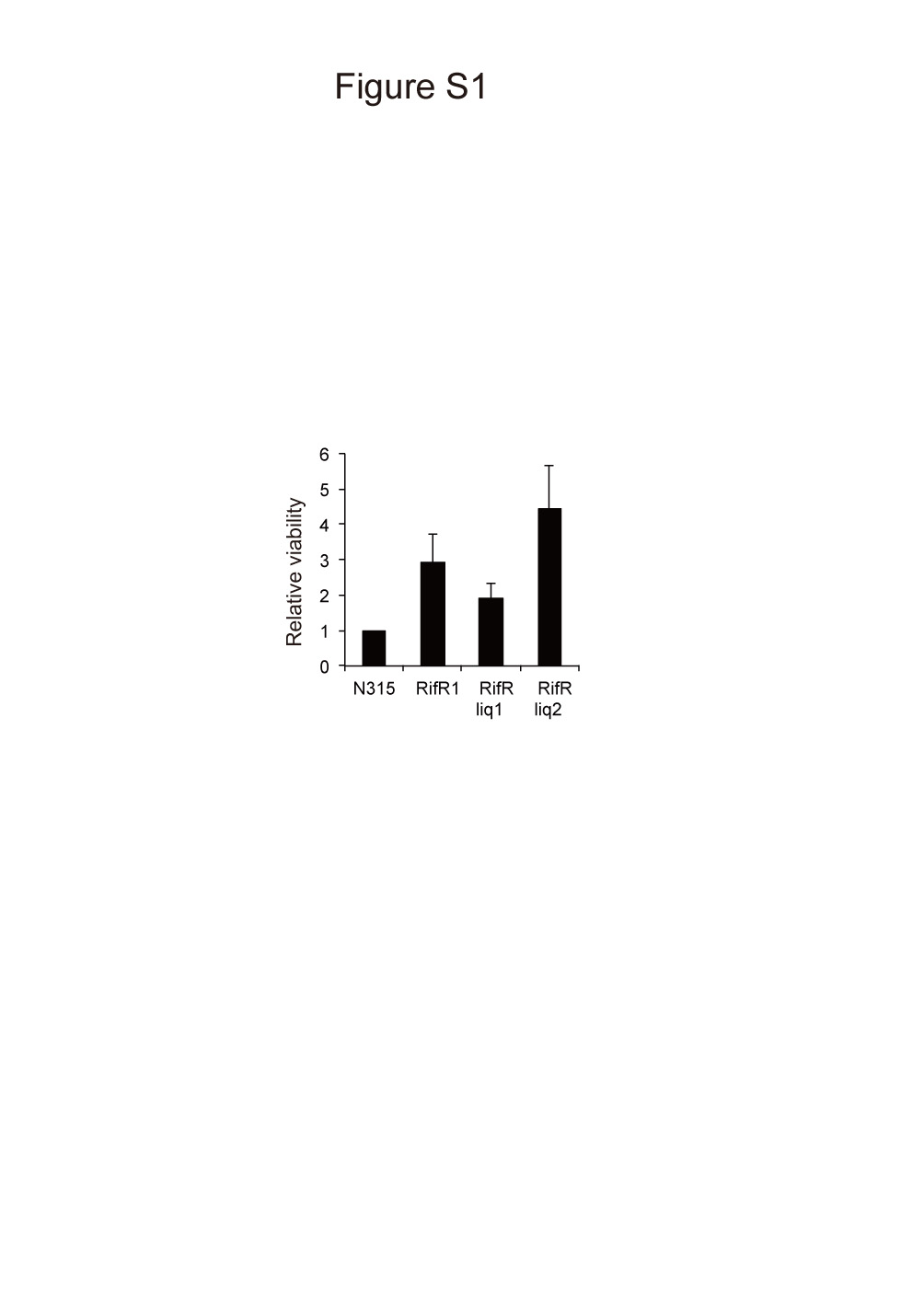

Supplement: FIGURE S1 — Viability of rifampicin resistant mutants isolated from stationary phase liquid cultures (Rif liq1, Rif liq2) in biofilm. Relative viabilities compared to wild-type (N315) are shown with standard errors (n = 3). [file Image_1.JPEG]

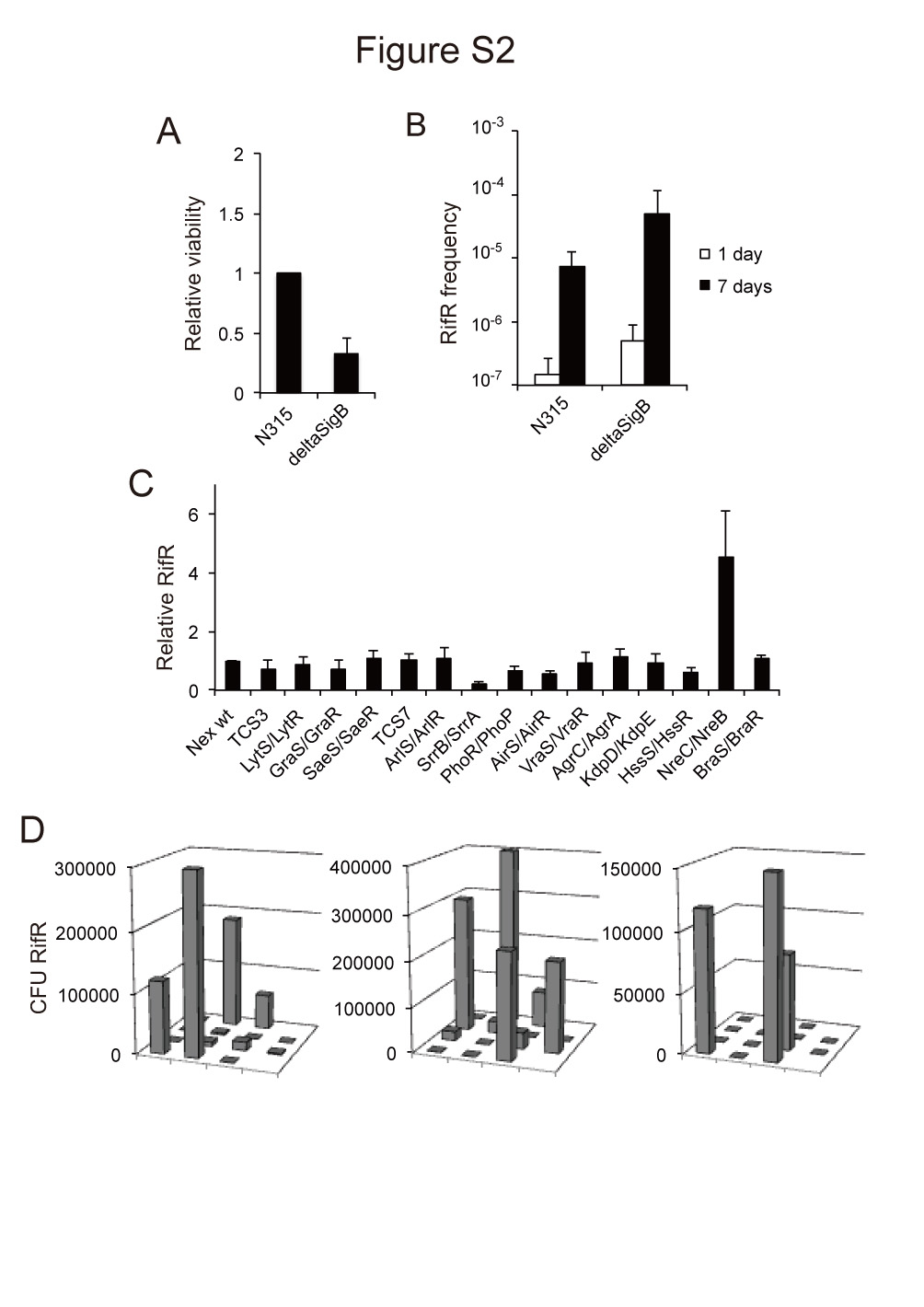

Supplement: FIGURE S2 — Involvement of stress response systems in RifR accumulation. (A,B) SigB is important for viability in biofilm, but dispensable for the accumulation of RifR cells. (C) Relative number of RifR mutants after 7 days incubation is shown. The ΔSrrA/B mutant contained very few RifR cells, while ΔNreB/C mutant contained increased number of RifR cells. (D) Distribution of the RifR cells in the biofilm of ΔNreB/C. [file Image_2.JPEG]

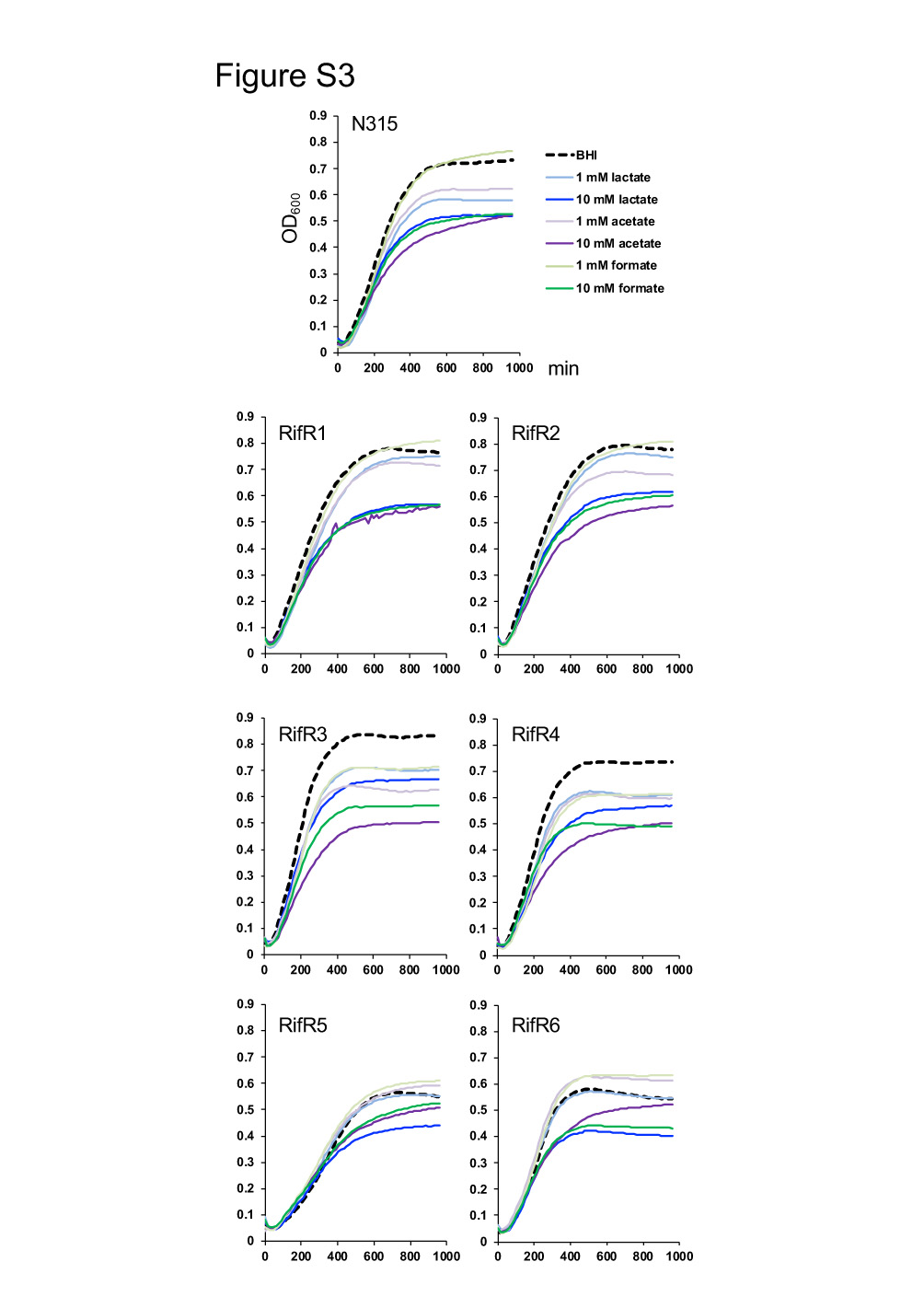

Supplement: FIGURE S3 — Effects of organic acids on the growth of rifampicin resistant mutant. Growth curves of each strain in the same experiment of Figure 5. [file Image_3.JPEG]
